# Incorporation of gantry angle correction for 3D dose prediction in intensity-modulated radiation therapy

**DOI:** 10.1093/jrr/rrv008

**Published:** 2015-03-04

**Authors:** Iori Sumida, Hajime Yamaguchi, Hisao Kizaki, Keiko Aboshi, Mari Tsujii, Yuji Yamada, Masashi Yagi, Kazuhiko Ogawa

**Affiliations:** 1 Department of Radiation Oncology, Osaka University Graduate School of Medicine, 2-2 Yamada-oka, Suita, Osaka, 565-0871, Japan; 2 Department of Radiation Oncology, NTT West Osaka Hospital, 2-6-40 Karasugatsuji, Tennoji-ku, Osaka, 543-8922, Japan

**Keywords:** MLC, IMRT, prediction, QA, non-gap test

## Abstract

Pretreatment dose verification with beam-by-beam analysis for intensity-modulated radiation therapy (IMRT) is commonly performed with a gantry angle of 0° using a 2D diode detector array. Any changes in multileaf collimator (MLC) position between the actual treatment gantry angle and 0° may result in deviations from the planned dose. We evaluated the effects of MLC positioning errors between the actual treatment gantry angles and nominal gantry angles. A gantry angle correction (GAC) factor was generated by performing a non-gap test at various gantry angles using an electronic portal imaging device (EPID). To convert pixel intensity to dose at the MLC abutment positions, a non-gap test was performed using an EPID and a film at 0° gantry angle. We then assessed the correlations between pixel intensities and doses. Beam-by-beam analyses for 15 prostate IMRT cases as patient-specific quality assurance were performed with a 2D diode detector array at 0° gantry angle to determine the relative dose error for each beam. The resulting relative dose error with or without GAC was added back to the original dose grid for each beam. We compared the predicted dose distributions with or without GAC for film measurements to validate GAC effects. A gamma pass rate with a tolerance of 2%/2 mm was used to evaluate these dose distributions. The gamma pass rate with GAC was higher than that without GAC (*P* = 0.01). The predicted dose distribution improved with GAC, although the dosimetric effect to a patient was minimal.

## INTRODUCTION

Intensity-modulated radiation therapy (IMRT) is designed to closely match the dose delivered to a target while minimizing exposure to normal tissue. To ensure that complex isodose distributions are delivered precisely, it is necessary to perform quality assurance (QA) for the multileaf collimator (MLC) and beam output, and to ensure that the QA results are within the suggested predefined tolerances [1–3]. One source of dose errors with step-and-shoot IMRT is the difference between the actual positions and the expected positions of MLC leaves [4], whereas that for sliding window IMRT is the difference in the motion and velocity of MLC leaves [5]. Verifying the absorbed dose measurements and isodose distributions is generally performed as patient-specific QA with a water-equivalent solid phantom before beginning radiation treatment.

It is generally preferable to use a phantom that is shaped as closely as possible to the dimensions of a particular patient. Its water-equivalent density makes it possible to verify the accuracy of beam delivery. When dose errors are found in the QA results, their effects on actual patients, if any, are difficult to predict. Recent dose prediction methods for 3D dose distributions in patients have used the measured data generated by pre-treatment QA. A currently used commercial prediction method (SNC, Melbourne FL, USA) involves a planned dose perturbation (PDP) algorithm [6]. Several studies have evaluated its accuracy and effectiveness [7, 8].

We previously proposed and validated a 3D dose prediction algorithm based on measurements made in two dimensions [9]. Our dose prediction algorithm used data from measurements made with irradiation at a gantry angle of 0° in the coronal plane with 2D diode detector arrays in each beam and created a relative dose error map for comparisons with the dose planning map. This relative dose error was then applied to 3D dose grids. However, the beam angle for an actual radiation treatment is different from that used for QA measurements.

Using an MLC QA non-gap test, we also previously showed that pixel intensity at the MLC leaf abutment varied significantly with any changes in gantry angle [10]. These findings demonstrated that the predicted dose distribution may differ, depending on whether the relative dose change at the MLC leaf abutment was factored in.

The aim of the current study was to predict 3D dose distributions more accurately using a gantry angle correction (GAC) for the QA results with a non-gap test. We generated numerical factors for a GAC from the QA results for MLC leaves performed weekly using an electronic portal imaging device (EPID).

## MATERIALS AND METHODS

### Field size verification at various gantry angles

For this study, we employed a 6-MV and a 10-MV linear accelerator (ONCOR Impression Plus; Siemens Medical Systems, Concord CA, USA). To acquire portal images, we used an EPID (Siemens OPTIVUE 1000, Siemens Medical Systems, Concord CA, USA). The detector had 1024 × 1024 pixels, with a spatial resolution of 0.40 mm/pixel. Because the pixel intensity at an MLC leaf abutment is affected by the MLC leaf position, field size analyses were performed at a range of gantry angles. We irradiated the EPID using field sizes of 5 cm × 20 cm, 10 cm × 20 cm, and 20 cm × 20 cm with gantry angles of 15° increments over a 360° rotation. The source–image distance (SID) was 150 cm. For each irradiation, we inserted a reticule with two orthogonal tungsten wires (XRETIC, Siemens Medical Systems) into the shadow tray as a substitute for a mechanical isocenter. Because the physical center of the EPID (rows: 511, columns: 511) was not exactly matched to the cross-point of the reticule on an EPID image, we measured the shift data (which consisted of rotational and translational offsets) by matching the physical center of EPID with the projected image of the tungsten wires. The reticule was used to correct the sag and translation of the EPID with respect to the isocenter at each gantry angle. The field size was defined in each leaf pair by measuring the full width at half maximum in pixel intensity. Validation of the measured field size by the EPID had already been commissioned, by comparing it with film measurements (data not shown).

### Non-gap test reproducibility

**Fig. 1.**
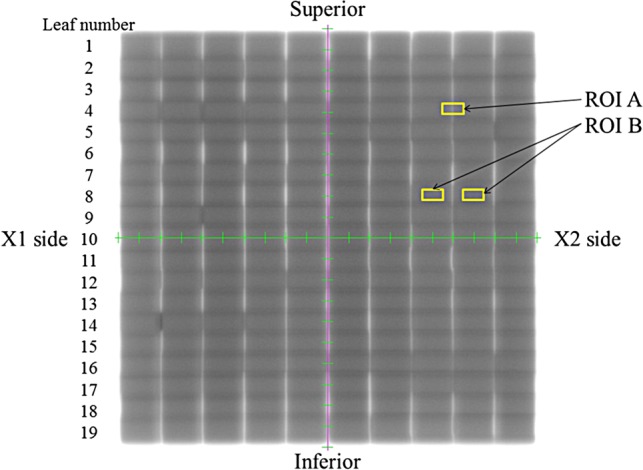
Sample composite image for a non-gap test at a gantry angle of 0° using an EPID. Scales in green and cyan show the *x* and *y* axes at 1-cm intervals. The region of interest (ROI) in yellow is 10 mm wide and 5 mm high. ROI A is located on the MLC leaf abutment. The B ROIs are located on the open fields. Abutments in white indicate a lower dose and those in black indicate a higher dose as compared with the dose to the open field.

To simulate a 20 cm × 20 cm open field, we sequentially exposed a rectangular field of 2 cm × 20 cm to one monitor unit (MU) onto the EPID 10 times at 2-cm intervals without a leaf gap. We acquired all EPID images at an SID of 150 cm using a 6-MV photon beam. For each image, we acquired 10 images using our in-house software and created a composite image. Figure 1 shows a sample image at a gantry angle of 0° for this test. We set the integral signal in the small region of interest (ROI; 10 mm × 5 mm) to Positions A (MLC leaf abutment: gap) and B (its neighbor: open field). Once one of the ROIs was positioned on the composite image for either Position A or B, the others were automatically defined based on a leaf abutment gap interval of 2 cm and a leaf width of 1 cm. Mean pixel values within the ROIs for A and B were determined.

For Region B, the mean pixel value from both sides of the gap region was determined and used to remove radiation field variations. The pixel value at Position B was used as background intensity. The EPID image pixel values at Position A were divided by an open field image at Position B to reduce possible variations in beam output and symmetry and to minimize the effects of local EPID response variations. The ratio of pixel values (A/B × 100) at each MLC abutment position was used to determine regions with an underdose, an overdose or a flattened dose. A pixel ratio at Position A of >100 was assumed to indicate overdosing in the leaf gap, and a ratio of <100 was assumed to indicate underdosing. We performed these procedures 10 times at a gantry angle of 0° to evaluate reproducibility.

### Conversion between pixel intensities and absolute doses at each MLC abutment

**Fig. 2.**
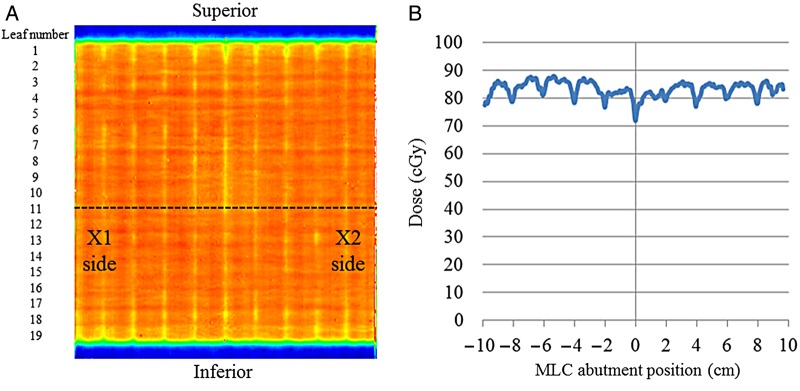
**(A)** Dose distribution for a non-gap test at a gantry angle of 0° using film. **(B)** The dose profile at leaf number 11 is shown by the dotted line in (A).

We performed the non-gap test at a gantry angle of 0° using radiochromic film (Gafchromic^TM^ EBT2, International Specialty Products, Wayne NJ, USA) that was inserted in the coronal plane (SAD: 100 cm, depth: 10 cm) in a water-equivalent solid phantom. Each rectangular field of 2 cm × 20 cm received 100 MU to attain a satisfactory film density for conversion to the absorbed dose. After irradiation, the film was scanned with a flatbed scanner (Perfection V700, Seiko EPSON Corp., Nagano, Japan) with 48-bit color and 150 dots per inch. When film density was converted to the absorbed dose, we chose a pixel value in the red channel of 16 bits because of its high dose sensitivity [11]. Figure 2 shows a representative dose distribution with the non-gap test based on film measurements and a sample dose profile at leaf number 11 in the left-to-right direction.

We calculated the relative dose at each abutment position as compared with the mean dose at the neighboring positions in the center of the 2-cm open field. A mean ratio for pixel values measured by the non-gap test performed 10 times was used for EPID measurements. Pearson correlation analysis was used to assess correlations between the relative doses derived from the film measurements and the ratios of pixel values derived from the EPID measurements for each abutment, which generated an approximation formula, from which we determined its slope.

### Correction factor for gantry rotation based on the non-gap test

We performed the non-gap test using an EPID at gantry angles of 0, 45, 90, 105, 135, 180, 225, 255, 270 and 315°. The total number of MLC abutment positions was 171 (nine locations in the lateral direction at 2 cm apart multiplied by 19 locations in the longitudinal direction at 1 cm apart, which was the same as the MLC leaf width) in each composite image. A correction factor at each abutment position for the other gantry angles was determined based the relative value against the intensity at the same abutment location with a gantry angle of 0°. We then created 2D look-up tables for the correction factors for all gantry angles. We converted all correction factors based on pixel intensity to those based on dose by using the slope derived from the approximation formula. We determined the correction factor at each abutment position (except for the measured abutment position of 2 cm apart) by linear interpolation between the two correction factors for neighboring abutments.

### GAC factor adaptation

We delivered treatment at five gantry angles (45, 105, 180, 255 and 315°) using a 10-MV X-ray beam and with a leaf width of 10 mm for step-and-shoot delivery. A total of 15 prostate cancer patients who were being treated with IMRT were included in this study. After a physician delineated the contouring of the clinical target volume (CTV), with the prostate as the target and the bladder and rectum as critical organs, a medical physicist created the plan with Treatment Planning System (TPS) (XiO, ELEKTA, Stockholm, Sweden). We performed dose calculations using a grid size of 2 mm. A mean dose of 78 Gy over 39 fractions was prescribed for the prostate planning target volume (PTV) for all these patients.

For dose distribution analysis, we performed per-beam QA using a 2D detector diode array (MAPCHECK, Sun Nuclear Corporation, Melbourne FL, USA) for one fraction of the treatment dose for each patient. We calculated the dose in a 30 × 30 × 30 cm^3^ thick solid water phantom at a gantry angle of 0° (rather than the actual treatment gantry angle) by assuming that there was no drift in the MLC leaf stop positions with different angles. The source-to-detector distance was 100 cm and the depth was 10 cm. After performing the dose calculations with a 1-mm grid, we exported the dose distribution in the coronal plane for each beam at a depth of 10 cm (in text format from TPS). We exported the Digital Imaging and Communications in Medicine–Radiation Therapy (DICOM–RT) plan, the DICOM–RT structure set, and the DICOM–RT dose for each beam in order to evaluate the dose–volume indices in the treatment plan for each patient.

After completing the per-beam QA using a 2D detector diode array for 15 patients, we created a 2D error map of calculated vs measured dose distributions and adapted this to the 3D DICOM–RT dose grid for each beam. The details for a 2D error map back-projected onto a 3D dose grid were described in a previous study [9].

**Fig. 3.**
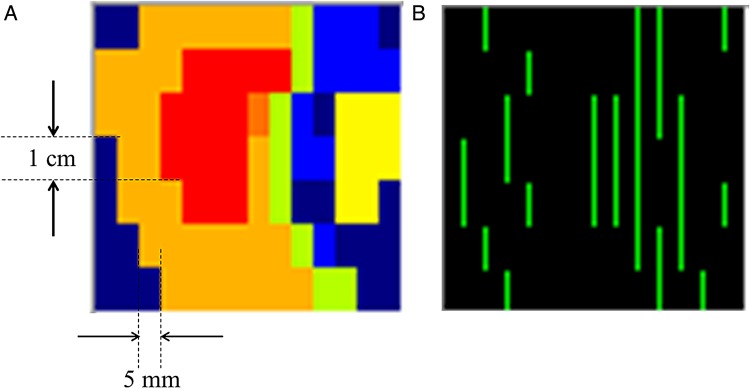
**(A)** Sample segmented intensity map. Red color indicates that the irradiated area had received a high intensity dose and blue color indicates that the irradiated area had received a low intensity dose. **(B)** MLC abutment detected image. Vertical lines in green indicate the detected MLC leaf edge.

We exported a segmented intensity map with the abutment positions of each MLC leaf pair after MLC leaf segmentation by TPS so as to apply the GAC factor to each beam. The grid resolution of the segmented intensity map was 1 mm. Figure 3 shows a representative segmented intensity map and the abutment positions detected, based on the differences in intensity between the neighboring segments. The abutment position of each leaf pair was determined by scanning the intensity profile at 1-mm intervals along the MLC leaf motion and detecting the different intensities at the edge. Using the GAC factor for each gantry angle described in the previous section, the corrected value at each abutment position per beam was determined. When adapting a GAC, we determined the final correction factor from the GAC factor in addition to the relative dose error of a 2D error map.

### Comparisons of dose distributions with or without GAC

**Fig. 4.**
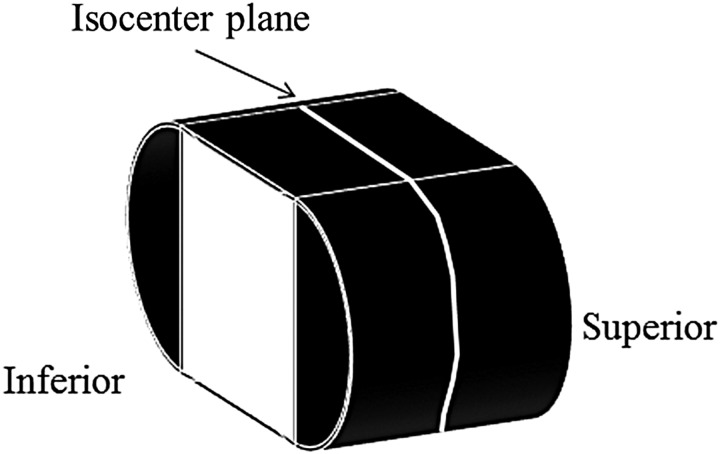
Measurement locations for a dose distribution. One sheet of film was inserted into the phantom's isocenter plane in the cranio–caudal direction.

**Fig. 5.**
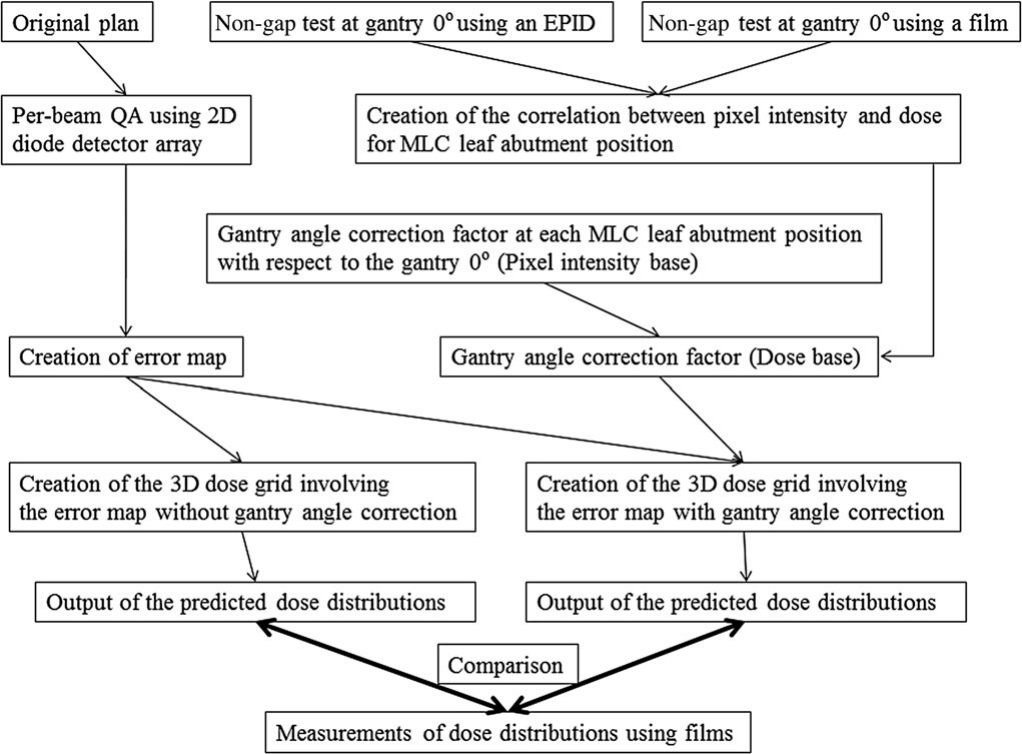
Dose verification procedures for our proposed method with and without GAC.

We compared dose distributions with or without GAC in 15 patients by verifying the isodose distributions in the axial plane using a film and a verification phantom (I'm*RT* Phantom, IBA Dosimetry, Schwarzenbruck, Germany). We inserted one sheet of radiochromic film (Gafchromic^TM^ EBT2, International Specialty Products, Wayne NJ, USA) in the isocenter plane in the cranio–caudal direction, as shown in Fig. 4. After overlaying treatment beams onto this phantom, we determined two patterns of predicted dose distributions in the isocenter plane by employing our dose prediction method, and incorporating the 2D error map in the 3D dose grid with and without GAC. We then compared the two predicted dose distributions with the measured film dose distributions, using the dose distribution from film measurements as a reference. The procedures used for these comparisons are shown in Fig. 5.

To verify isodose distributions, three different dose evaluations—relative dose difference, distance to agreement, and percent pass rate of gamma function (3%/3 mm and 2%/2 mm criteria with a 10% low-dose threshold)—were used [12, 13]. The predicted dose distributions were evaluated by the gamma pass rate using Student's *t*-test for statistical comparisons (with *P* < 0.05 considered as significant).

### Dose evaluations for a target and normal tissues with and without GAC

For dose evaluations with dose–volume indices in a target, we calculated D_98%_ to the CTV and the PTV as a minimum dose, D_2%_ to the CTV and the PTV as a maximum dose, the mean dose to the CTV and the PTV, and D_95%_ to the PTV and expressed these values in Gy. We chose D_98%_ and D_2%_ based on the dose specification protocol of ICRU Report 83 [14]. For the organs at risk (OARs), the percentage of rectal tissue that received 65 Gy (V_65Gy_) and the percentage of bladder tissue that received 40 Gy (V_40Gy_) were determined. A physician set rectal dose constraints of V_65Gy_ < 17% and V_40Gy_ < 35%, and bladder dose constraints of V_65Gy_ < 25% and V_40Gy_ < 50%, based on a previous report [15], and these were used to develop the planning goal. For the 15 patients enrolled in this study, we compared the dose–volume indices for the three patterns: the predicted doses with and without GAC, and the original plan (using Student's *t*-test for statistical comparisons). The significance level was set at *P* < 0.05.

## RESULTS

### Field size verification for different gantry angles

**Fig. 6.**
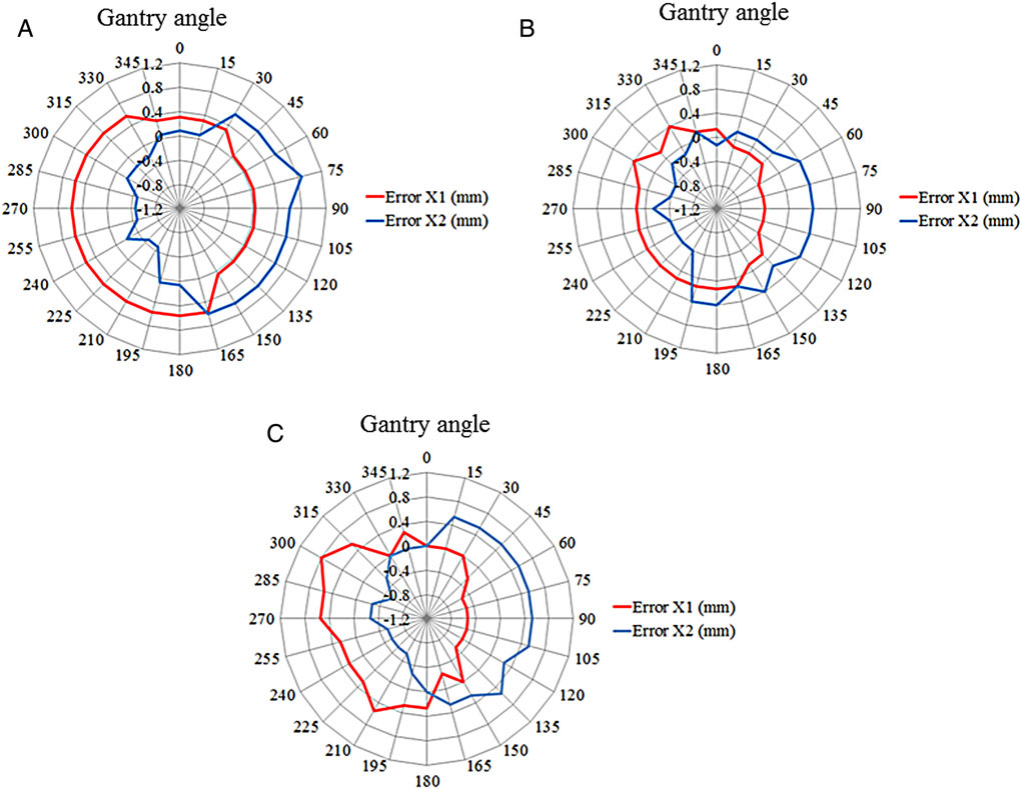
Field size errors for three nominal field sizes of **(A)** 5 cm × 20 cm, **(B)** 10 cm × 20 cm, and **(C)** 20 cm × 20 cm at the center MLC leaf. Nominal field sizes of each side (X1 and X2) were 2.5 cm, 5 cm and 10 cm for (A), (B) and (C), respectively. Positive values indicate a size larger than the nominal field size. Negative values indicate a size smaller than the nominal field size.

Figure 6 shows the field size errors for three nominal field sizes of 5 cm × 20 cm, 10 cm × 20 cm, and 20 cm × 20 cm. The nominal field sizes of each side (X1 and X2) were 2.5 cm, 5 cm and 10 cm for (A), (B) and (C), respectively. A positive value indicated a field size larger than the nominal. A negative value indicated a field size smaller than the nominal.

For the field size of 5 cm × 20 cm, the size changes were 0.31 mm and 0.09 mm on the X1 and X2 sides at a gantry angle of 0°, 0.04 mm and 0.60 mm at a gantry angle of 90°, 0.57 mm and 0.06 mm at a gantry angle of 180°, and 0.57 mm and −0.47 mm at a gantry angle of 270°. For the field size of 10 cm × 20 cm, the changes were 0.14 mm and −0.14 mm on the X1 and X2 sides at a gantry angle of 0°, −0.40 mm and 0.40 mm at a gantry angle of 90°, 0.14 mm and 0.40 mm at a gantry angle of 180°, and 0.14 mm and −0.14 mm at a gantry angle of 270°. For the field size of 20 cm × 20 cm, the changes were 0.00 mm and 0.00 mm on the X1 and X2 sides at a gantry angle of 0°, −0.53 mm and 0.53 mm at a gantry angle of 90°, 0.27 mm and 0.00 mm at a gantry angle of 180°, and 0.54 mm and −0.27 mm at a gantry angle of 270°.

Due to the effects of gravity, for any nominal field size, there was the same tendency for the opening size of the X2 side to increase over angles of ∼15–165°, and for the opening size of the X1 side to narrow to the same extent over the same gantry angle range. In contrast, the opening size of the X1 side was increased or stable from 180° to 330°, and the opening size of the X2 side was decreased or stable over the same gantry angle range.

### Non-gap test reproducibility

**Fig. 7.**
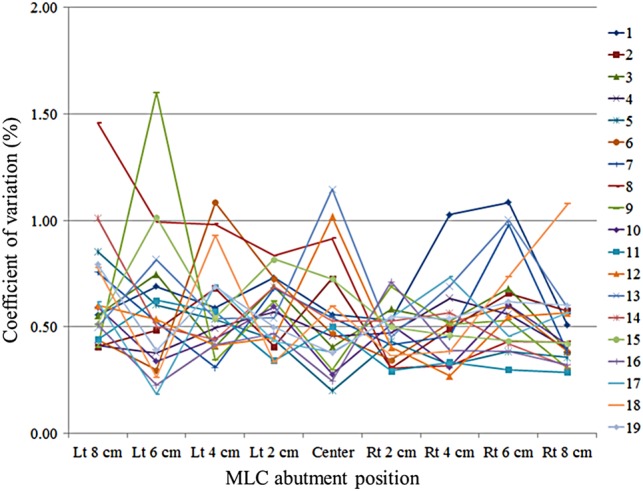
Coefficients of variation measured 10 times at each MLC abutment position for 19 leaves. The *x*-axis shows the MLC abutment positions ranging from 8 cm to the left to 8 cm to the right at 2-cm intervals, and the *y*-axis shows the coefficients of variation at the MLC abutment positions measured 10 times. Individual leaf numbers are represented by different colored lines.

We performed the non-gap test 10 times at a gantry angle of 0° using an EPID. Figure 7 shows the coefficients of variation for the pixel value ratios at each MLC abutment position. The mean ± SD of these coefficients of variation was 0.55 ± 0.23%, and ranged between 0.18% and 1.60% (95% CI: 0.1%–1.0%).

### Conversions between pixel intensities and absolute doses at each MLC abutment

**Fig. 8.**
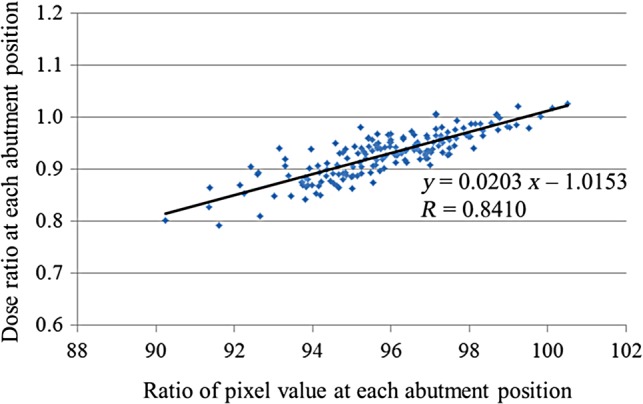
Relationship between dose ratios and pixel value ratios at each MLC abutment position. The approximation formula is shown in the figure. The correlation coefficient was 0.8410, which indicated a strong correlation.

We measured the relative dose ratios at each MLC abutment using a film. Figure 8 shows the correlation between the pixel value ratios and the relative dose ratios at each MLC abutment. The approximation formula was:

(1) Relativedoseratio=0.0203×Ratioofpixelvalue−1.0153

There was a statistically significant correlation between the pixel value ratios and relative dose ratios (*r* = 0.8410; *P* < 0.001). The slope of this curve was 0.0203, which indicated that when the pixel value ratio changed by a value of 1, the relative dose ratio increased by 2.03%. When the change in the pixel value ratio was negative, the relative dose ratio decreased. The *y*-intercept value of –1.0153 was not employed because we used the relative change in the pixel value ratios at different gantry angles against those at a gantry angle of 0°.

### Correction factors for gantry angles based on non-gap test results

**Fig. 9.**
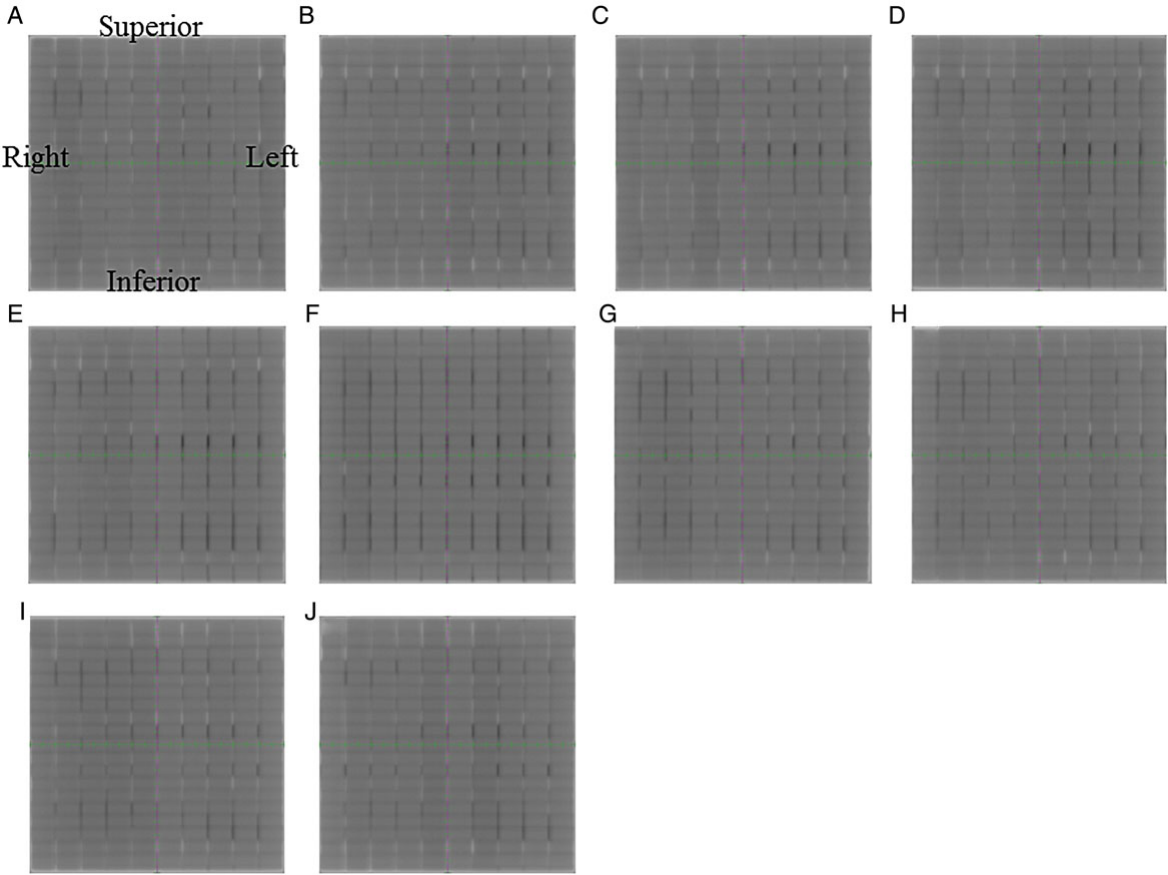
Composite images of the non-gap test at gantry angles of **(A)** 0, **(B)** 45, **(C)** 90, **(D)** 105, **(E)** 135, **(F)** 180, **(G)** 225, **(H)** 255, **(I)** 270 and **(J)** 315 degrees. MLC abutments in white indicate a lower dose as compared with the dose to the open field, and those in black indicate a higher dose as compared with the dose to the open field.

**Fig. 10.**
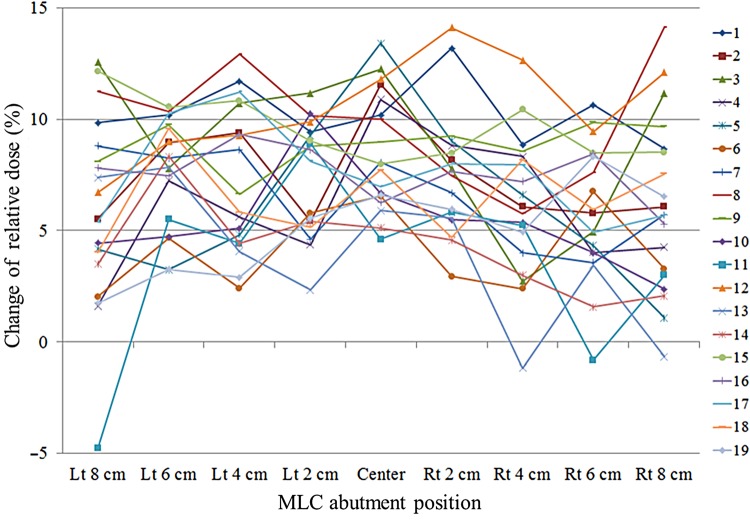
Example of the changes in relative dose at a gantry angle of 180° as compared with those at a gantry angle of 0° for 19 leaves. The *x*-axis shows the MLC abutment positions ranging from 8 cm to the left to 8 cm to the right at 2-cm intervals, and the *y*-axis shows the changes in relative dose. Individual leaf numbers are represented by different colored lines.

We determined correction factors for MLC abutment positions for gantry angles of 0, 45, 90, 105, 135, 180, 225, 255, 270 and 315°, by normalizing each factor to the value at a gantry angle of 0°. Figure 9 shows composite images over the entire gantry angle range. The pixel intensity at each MLC abutment position varied with the gantry angle. At a gantry angle of 180° in particular, there were overdose values at Leaf Number 12, whereas there were none at a gantry angle of 0°. As an example, Fig. 10 shows the changes in relative dose at a gantry angle of 180° as compared with a gantry angle of 0°. The changes in relative dose at any abutment position for all leaf pairs had positive values of 6.97 ± 3.21% (mean ± SD).

### Comparisons of dose distributions with or without GAC by film analysis

**Fig. 11.**
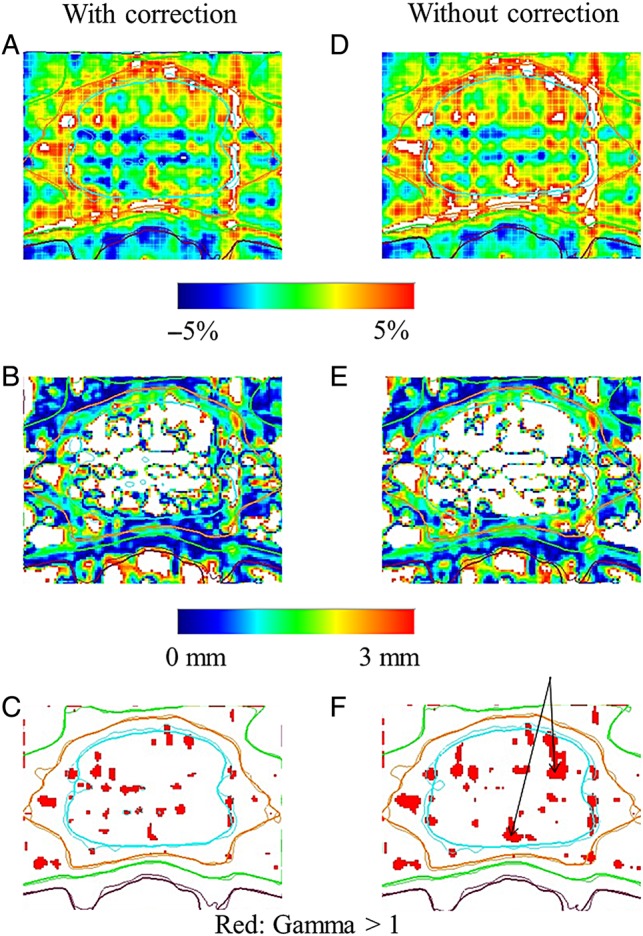
Comparisons of dose distributions with and without GAC. Panels (**A**), (**B**) and (**C**) are comparisons of dose distributions with GAC and film measurements. Panels (**D**), (**E**) and (**F**) are comparisons of dose distributions without GAC and film measurements. The film dose distribution was used as a reference. For dose differences, the error range was 5% in (A) and (D). Panels (B) and (E) show the mean distances to agreement with an error range of 3 mm. Panels (C) and (F) show the gamma distributions. A gamma value of >1 is shown in red. Black arrows in (F) indicate the regions where the segment intensities were very different.

Dose distributions with or without GAC at the isocenter plane are compared in Fig. 11 for one of the 15 patients in this study. Using the film dose distribution as a reference, there was closer agreement when using GAC (Fig. 11A) than without using GAC (Fig. 11D). The predicted dose distribution without GAC underestimated that derived from a film measurement. The larger dose differences that resulted in an overdose region are shown in Fig. 11D. There was a tendency for these overdose regions to occur at the interface between the target and an OAR, probably because the drop-off in intensity between the target and an OAR was high.

For the distance to agreement, we matched the predicted isodose distributions both with and without GAC to the measured isodose distributions within 3-mm tolerance in Fig. 11B and E, respectively. Figure 11B illustrates closer matching as compared with Fig. 11E. For the gamma analysis with the tolerance criteria (3%/3 mm) for the percent pass rates of the predicted dose distribution with GAC and the film measurements in Fig. 11C, the result was 94.3%. The result for the equivalent analysis of the predicted dose distribution without GAC and the film measurements in Fig. 11F)was 91.7%. With regard to the tolerance criteria (2%/2 mm), the gamma pass rates were 79.6% and 75.4%, respectively. Again, the regions with gamma values of >1 (in red) were primarily located at the interface between the target and an OAR, where the segment intensity dropped sharply. Table 1 summarizes the gamma pass rates for the 15 cases evaluated in this study. The mean gamma pass rates with and without GAC under the 3%/3 mm criteria were significantly different at, respectively, 94.7 ± 2.9% and 93.3 ± 3.5% (*P* < 0.01). The mean gamma pass rates with and without GAC under the 2%/2 mm criteria were significantly different at 83.4 ± 5.4% and 81.4 ± 6.0% (*P* < 0.01), respectively.

**Table 1.** Gamma pass rates with 3%/3 mm and 2%/2 mm criteria for 15 cases

CASE	3%/3 mm tolerance		2%/2 mm tolerance	
	With GAC	Without GAC	With GAC	Without GAC
1	95.4%	93.8%	85.9%	84.0%
2	97.6%	97.2%	90.3%	89.4%
3	96.6%	95.3%	86.1%	83.2%
4	97.6%	97.0%	89.2%	87.3%
5	97.1%	96.0%	88.5%	87.5%
6	97.8%	96.7%	88.7%	87.0%
7	91.9%	91.0%	80.7%	78.2%
8	95.9%	94.7%	83.3%	82.4%
9	94.3%	91.7%	79.6%	75.4%
10	95.2%	94.8%	84.2%	83.4%
11	91.9%	89.1%	77.3%	74.2%
12	94.9%	94.0%	83.6%	82.6%
13	95.9%	94.3%	86.1%	84.1%
14	91.3%	87.8%	74.9%	71.0%
15	87.6%	85.8%	72.8%	70.7%

GAC = gantry angle correction.

### Comparisons of dose–volume indices for 15 cases

**Fig. 12.**
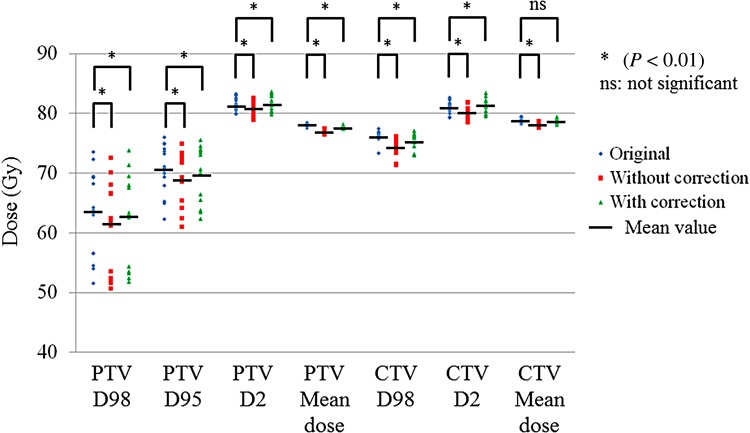
Dose–volume indices for the PTV and the CTV in three patterns of the predicted doses with GAC, without GAC, and the original plans. Solid black bars represent mean values. Student's *t*-tests were used to compare the original plans with plans with or without GAC (*P < 0.01: significant difference; ns: not significant).

The dose–volume indices for the PTV and the CTV are shown in Fig. 12. Using the data from the original plans as reference values, we compared the predicted doses with and without GAC. All dose–volume indices with GAC were closer to those of the original plan compared with those without GAC. Except for the dose–volume index of the mean dose for the CTV, we found significant differences between the original plans and the predicted doses with or without GAC (*P* < 0.01).

**Fig. 13.**
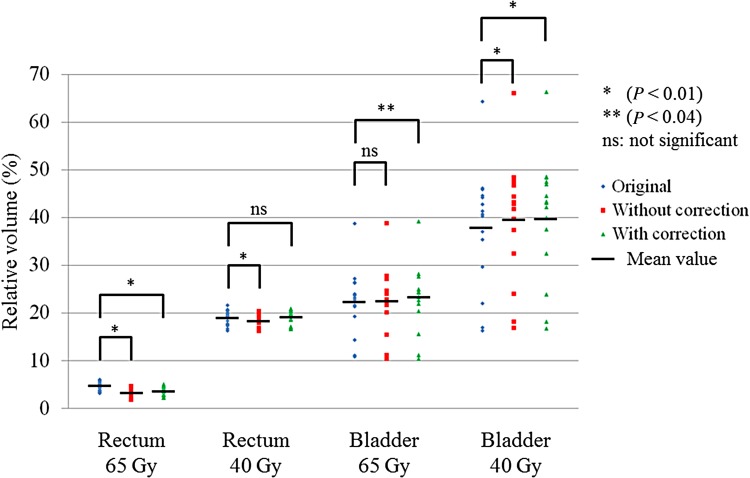
Dose–volume indices for the rectum and bladder among three patterns of the predicted doses with GAC, without GAC, and the original plans. Solid black bars represent mean values. Student's *t*-tests were used to compare the original plans with those with or without GAC (*P < 0.01, **P < 0.04: significant difference, ns: not significant).

The dose–volume indices for the rectum and bladder are shown in Fig. 13. V_65Gy_ values for the rectum with or without GAC were significantly lower than those of the original plans (*P* < 0.01). V_40Gy_ values for the rectum without GAC were also significantly lower than those of the original plans (*P* < 0.01), although those with GAC were not significantly different compared with those of the original plans. Except for V_65Gy_ values for the bladder at the predicted doses without GAC, the other volume indices of V_65Gy_ and V_40Gy_ for the bladder with or without GAC were significantly higher than those of the original plans (*P* < 0.01 and *P* < 0.04, respectively).

## DISCUSSION

For field size verifications at different gantry angles, there was a tendency for MLC leaf positions to move due to the effects of gravity at gantry angles approximately between 15° and 165° and approximately between 180° and 330°. For gantry angles between 15° and 165°, the X1 and X2 sides of the MLC leaves moved inferiorly, so that the opening of the X1 side was smaller compared with that at a gantry angle of 0°, and the opening of the X2 side was wider compared with that at a gantry angle of 0°. In other words, the field size did not change, but the field port translated inferiorly over a distance of <0.5 mm. For gantry angles of ∼180–330°, the X1 and X2 sides of the MLC leaves also moved inferiorly. As shown in Fig. 9C and I (compared with A), there was almost no change in pixel intensity at any MLC abutment position.

However, the changes in field size at a gantry angle of 180° compared with those at a gantry angle of 0° for 5 cm × 20 cm, 10 cm × 20 cm and 20 cm × 20 cm were 0.23, 0.54 and 0.27 mm, respectively. Although these changes were small, they were all in the positive direction. This phenomenon occurs with the MLC motion mechanism incorporated in this type of Linac because of the double-focused MLC. Each MLC moves in an arc trajectory with respect to the target source; therefore, the field size is wider due to the weight of the MLC. Ultimately, there was a tendency for the pixel intensity at the MLC abutment to be greater at a gantry angle of 180° than that at a gantry angle of 0°, as shown in Fig. 9A and F. At several institutions where step-and-shoot IMRT is performed, the MLC bank root mean square error was found to depend on the gantry angles, which could be explained by the effects of gravity, even though the MLC does not move in an arc trajectory [16]. There is also a concern regarding the effects of gravitational displacement of leaf position for both step-and-shoot IMRT and conformal dynamic arc and intensity-modulated arc therapy [17–19].

With regard to the conversions between pixel intensities and absolute doses at each MLC abutment position, we confirmed the reproducibility of the non-gap test using an EPID with a mean coefficient of variation of 0.55%; pixel intensity must then be converted to the relative dose. Because the absolute dose at each MLC abutment position for the various gantry angles was measured with a film, it was difficult to place the film at gantry angles other than 0, 90, 180 and 270°. In contrast, the EPID faces the source so that it is easy to measure the dose at any gantry angle. In addition to difficult measurement conditions, many steps are required to convert film density to dose, which increases the uncertainty in the analysis, such as scan conditions for a film [20] and the self-development of film density [21]. As shown in Fig. 8, we found a strongly positive correlation (*r* = 0.8410) in our conversion between pixel intensities and relative doses. It should be noted that the pixel intensity at any abutment position may change with MLC leaf calibration. Conversions between pixel intensities and relative doses should be performed after MLC leaf calibration.

**Fig. 14.**
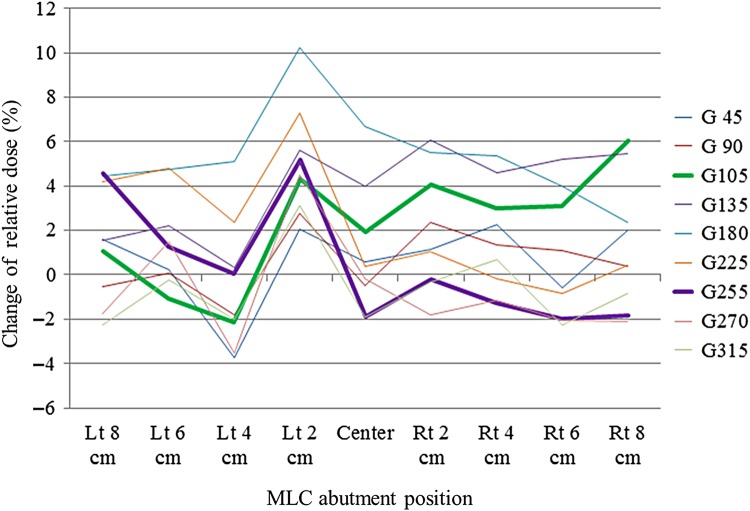
Changes in relative dose at each MLC abutment position with respect to the different gantry angles plotted against a gantry angle of 0° for a center leaf. The *x*-axis shows the MLC abutment positions ranging from 8 cm to the left to 8 cm to the right at 2-cm intervals. The *y*-axis shows the changes in relative dose. The different line colors represent different gantry angles. Thick lines are a gantry angle of 105° in green and an angle of 255° in purple, which are used as actual gantry angles for IMRT.

In this study, we selected prostate IMRT cases to evaluate the predicted doses with and without GAC and to validate the necessity of using GAC. Five fields at gantry angles of 45, 105, 180, 255 and 315° are commonly used in our department. The changes in relative dose at each MLC abutment position with respect to the various gantry angles as compared with that at a gantry angle of 0° for a center leaf are shown in Fig. 14. There was no tendency for the relative doses to change with respect to these gantry angles. In cases for which a medical physicist has planned to perform a non-gap test at gantry angles between 0 and 360° with 45° increments as periodic QA, gantry angles of 105° and 255° would not be included. We recognize the limitations posed by making calculations (derived from interpolations between the two neighboring gantry angles) of the relative dose changes at MLC abutment positions at these gantry angles used during actual treatments. Thus, it may be necessary to evaluate the performance of the non-gap test at those gantry angles used during actual treatments.

For film analysis to compare the predicted doses with or without GAC, the dose distributions with GAC were better matched to those of film measurements under both the 3%/3 mm and 2%/2 mm criteria. As shown in Fig. 11D, dose differences were relatively large at the periphery of a target, as well as inside the target. That is, the dose distribution on a film with GAC exhibited higher doses than those without GAC. In order to create a steep dose gradient between a target and an OAR, sharply different intensities are needed in neighboring segments. We derived gantry positions with dose errors from the high intensity modulated segments that were generated in a target and the surrounding normal tissues or at the interface between a target and the rectum from the beam's eye view at each beam.

For the 15 prostate IMRT treatments, the dose–volume indices for the PTV and the CTV with GAC were closer to those in the original plans as compared with those without GAC. The dose–volume indices for the rectum with GAC were also closer to those in the original plans as compared with those without GAC. Despite the differences in dose–volume indices, these values for the rectum may have been higher as a result of the intensities at MLC abutments (due to leaf calibration settings). In our previous study, we showed that the relative amount of change for a rectal volume index, particularly V_65Gy_, was large and depended on the leaf calibration settings used [9]. In contrast, both dose–volume indices for the predicted doses to the bladder were higher than those in the original plans. The bladder was located cranial to the target of interest, so segments were less intensity modulated in this region. Therefore, the dose–volume index for the V_65Gy_ for the bladder would not be influenced by the presence or absence of GAC. The dose–volume indices for the V_40Gy_ to the bladder with or without GAC were higher than those in the original plans. A reason for these differences may be that the Y-jaw opening was slightly wider than the nominal values–systematically higher than the original plan values, and as much as 40% higher. The mean values with or without GAC were nearly the same. When there is a systematic discrepancy such as this Y-jaw opening between measured and planned data, this discrepancy should be minimized. However, it is difficult to recognize these discrepancies during the commissioning process for IMRT, with the gamma passing rate under the common criterion of 3%/3 mm [2]. This is because the 3-mm criterion for distance-to-agreement passes the measured result at the steep dose gradient region. According to a treatment planning study for inducing systematic errors into the original plans, the beam output, MLC leaf gap width, tongue-and-groove effect, beam penumbra width, and MLC leaf transmission all affected the dose distribution, although the gamma passing rate was less sensitive (except for the beam output error) [22]. Therefore, we consider that one option may be to use the tighter criterion of ≤3%/2 mm during the commissioning process.

To predict dose distributions more accurately, we incorporated corrections for the actual treatment angles into the relative dose differences between the planned doses and the measured doses to a 2D diode detector array at a gantry angle of 0°. We generated GAC factors at MLC abutment positions using a non-gap test measured with an EPID. The predicted dose distributions with GAC were well matched to those of film measurements. However, GAC notwithstanding, the effect on the dose–volume indices to the target of interest and an OAR was minimal.

## FUNDING

Funding to pay the Open Access publication charges for this article was provided by Iori Sumida.
